# Nurses in the Male Wards of Public Asylums

**Published:** 1904-07-02

**Authors:** 


### Nurses in the Male Wards of Public Asylums.

The advisability of employing female nurses in the men's wards of our public asylums is coming to the front very quickly in Scotland; and this movement north of the Tweed is almost certain to mean that it will spread to some extent in England also. For 20 years or more it has been customary to employ nurses in the men's infirmary wards of several of our English larger county asylums; and in some instances these nurses are the wives of the male attendants. That is they have been trained as nurses in the women's wards of the asylum, but having married and lost their posts in the women's wards, have been subsequently taken on in the men's wards. It can hardly be said that the step has commended itself to the majority of English medical superintendents at present, whatever may be the extent of conversion that awaits them in the future.

In at least one English asylum the system was tried in other than the infirmary wards, and speedily broke down, giving place to the usual order of affairs; but this failure may have been due to the method in which the innovation was carried on, and not necessarily to any inherent weakness in the system itself. At the same time objections will occur to everyone familiar with the inner life of our large county asylums. Among others we may mention sudden outbreaks of violence on the part of a patient or patients, and some of our older medical superintendents would think twice before they subjected themselves or their nurses to the consequences of such outbursts. Then there are the bathing arrangements. An insane patient can hardly ever be trusted in the bath-room by himself. In wards managed by nurses how are the 30 or 40 or 50 patients to be bathed weekly or oftener? Again, supposing that the staff in a ward be composed partly of attendants and partly of nurses, then the following questions would at once arise. Where are the nurses to sleep? Where are they to take their meals? Are they to be considered part of the head nurses' staff or part of that of the head attendant? If the former, is the head nurse to visit the male wards? And who would be in charge of the ward—an attendant or a nurse? Of course these objections may be put down as more or less theoretical, and some of them would be non-existent if the nurses were to be supplementary to the male staff, and not substitutes for it. When mentioning these objections—and others will occur to every asylum medical officer—it must be borne in mind that in many things connected with the care and treatment of the insane theoretical objections have vanished before the practical results obtained by painstaking and far-seeing medical superintendents. We know how much distrust was shown when Gardiner Hill struck off the fetters of his patients in the Lincoln Asylum; a distrust which can still be read in the Minutes of the Committee of Visitors, and it may be that similar success will attend this innovation of nursing the insane as has attended the non-restraint system of treatment. We are here assuming that it will stray beyond the sick wards—in fact, it has already done so, and we do not know where it will stop or where it is intended that it will stop.

While pointing out some of the drawbacks of the extension of female nursing in asylums it is easy enough to see that certain advantages may accrue from its more limited adoption. Who is there who has not experienced the difficulty of making a man into a good sick-nurse? and who can help contrasting this difficulty with the ease with which a woman can be taught nursing? The man rarely rises to it in the same sense as the woman does. With the most praiseworthy intentions and with the many admirable qualities which asylum attendants possess, they rarely adapt themselves thoroughly to this side of their duties. The glory of the man can never be that of the woman. It is true also that male patients like being attended to by nurses; and they will often willingly obey a nurse when they would refuse to comply with the wish of an attendant. It is an old asylum saying that a refusal-of-food case should never be forcibly fed until he has declined to take food in the usual way from a nurse.

Then the structure of the asylum has something to do with the question. Where the ward or block can be treated as a distinct unit, the innovation would seem to be freer from the objections of a disciplinary nature than when this separation cannot be completely carried out; but it adds to the difficulty in case of violence among the patients, for it might be impossible to call in the aid of male attendants in time to prevent a serious accident. Naturally, nurses would not be placed in charge of wards where excited patients were known to be. But who can tell when the first uncontrollable outburst of temper will take place; and many asylum physicians will affirm that the more they see of so-called harmless lunatics the less they trust them.

At present we prefer to await the results of a wider and longer trial of the care of the male insane by nurses before we express any very decided opinion either for or against it. What we want is a definite, authoritative statement of the advantages growing out of and peculiar to the system, and proof that such advantages could not have been obtained by the employment of attendants, and that these advantages outweigh the manifest theoretical disadvantages of this employment of nurses beyond the nursing of a limited number of the insane sick. The mere expression of an opinion is not enough. An artist takes to the landscape what he paints there, and a medical superintendent with the best intentions of judicial balancing is not unlikely to be convinced that practice bears out his own theory.

